# A matter of time: Bateman's principles and mating success as count and duration across social strata in contemporary Finland

**DOI:** 10.1098/rspb.2023.1061

**Published:** 2023-07-12

**Authors:** Linus Andersson, Marika Jalovaara, Jan Saarela, Caroline Uggla

**Affiliations:** ^1^ Department of Social Research, University of Turku, Turku, Varsinais-Suomi, 20014 Finland; ^2^ The Swedish Institute for Social Research, Stockholm University, Stockholm, 10691, Sweden; ^3^ Åbo Akademi, Abo, Varsinais-Suomi, 20500 Finland; ^4^ Stockholm University Demography Unit (SUDA), Department of Sociology, Stockholm University, Stockholm, 106 91, Sweden

**Keywords:** serial monogamy, evolutionary demography, sexual selection, Bateman gradient, fertility, cohabitation

## Abstract

Bateman's principles heavily influence the understanding of human reproductive behaviour. Yet, few rigorous studies on Bateman's principles in contemporary industrialized populations exist. Most studies use small samples, exclude non-marital unions, and disregard recent insights on within-population heterogeneity in mating strategies. We assess mating success and reproductive success using population-wide Finnish register data on marital and non-marital cohabitations and fertility. We examine variability across social strata in the Bateman principles and analyse the mate count, the cumulated duration with a mate, and the association with reproductive success. Results support Bateman's first and second principles. Regarding Bateman's third principle, the number of mates is more positively associated with reproductive success for men than women, but this association is driven by ever having a mate. Having more than one mate is on average associated with lower reproductive success. However, for men in the lowest income quartile, having more than one mate positively predicts reproductive success. Longer union duration is associated with higher reproductive success, and more so for men. We note that sex differences in the relationship between mating success and reproductive success differ by social strata, and argue that mate duration may be an important component of mating success alongside mate count.

## 1. Introduction

Bateman's 1948 study of fruit fly mating concluded that variability in reproductive success (RS) was greater among males and that the difference was driven by the variance in the number of mates. The study was later crystallized into three principles: (1) males show greater variance in RS than females, (2) males show greater variance in mating success (MS), and (3) the positive association between MS and RS is stronger for males than females [1]. These principles were incorporated into parental investment theory [2] and epitomized as the Darwin–Bateman paradigm, which has been hugely influential [3]. The purported stronger positive link between MS and RS for males than females has been pivotal in advancing the view of distinct sex-differences in psychology and behaviour in both non-human animals and humans, and has guided assumptions of sex roles in choosiness and competition for mates [4].

Despite critiques of the methodology and theoretical foundations, the Bateman gradient continues to influence research on sex roles and sexual selection [5,6]. The critique has led to a re-evaluation of the role of anisogamy, and its role in the presumption of fitness pay-offs to coy females and competitive males. Conversely, females may benefit from mating with multiple males [7,8] and should not invariably favour parenting over mating effort just because costs of gestation and lactation have already been paid [8,9]. Other factors, such as the adult sex ratio, can alter pay-offs to mating versus parenting efforts to males and females alike (for reviews, see [10,11]). Recent cross-species meta-analyses question Bateman's third principle by showing a weak correlation between the degree of anisogamy and sexual selection [12]. Other studies—which measure opportunity for selection, opportunity for sexual selection, and the Bateman gradient rather than the selection of traits—have found a stronger positive relationship between MS and RS for females than males [13], and an equally strong gradient among both sexes [14]. Moreover, the measurements of Batemans first and second principle—opportunity for selection *(I)* and opportunity for sexual selection (*I*_S_)—have been questioned [15–18]. While stochasticity feeds into both *I* and *I*_S_, its magnitude correlates with environmental factors. Random chance is greater at high levels of operational sex ratios (OSR), at smaller population sizes, and at lower mean levels of fitness [16], under conditions of high mate monopolization [18] and low mortality in reproductive ages [19].

### (a) The Bateman principles in humans

In humans, variance in both MS and RS tend to be higher among males than females [1] and several studies have found support for Bateman's third principle, i.e. that there is a stronger relationship between MS and RS among males. Analyses from eighteenth century Utah [20], and eighteenth and ninteenth century Finland [20–22] have shown that multiple mates benefited male RS but not female RS. What we currently know about the Bateman gradient in humans is to a large extent based on such historical data from contexts in which women were not able to initiate divorce and where re-partnering was frequently the result of the death of a spouse. Furthermore, there is a paucity of studies that examine the Bateman principles in contemporary low-fertility societies [3]. Studies based on data from the twenty-first century include one from the US [23] and one from Sweden [24], both of which demonstrated a stronger association between MS and RS for men than women. However, these studies from contemporary industrialized populations [23,24] are based on survey material with documented limitations, including sample attrition and measurement error in male fertility [17,25].

An important limitation of several previous studies is the operationalization of MS as the number of spouses. In societies where a substantial share of mating occurs in non-marital cohabiting unions, this is both a conceptual problem and an empirical shortcoming. In most contemporary industrialized populations, serial monogamy is to a great extent driven by non-marital unions [26]. Consequently, estimates of Bateman's second and third principles may be severely distorted owing to under-estimation of MS by (non-marital) unions. Furthermore, while non-marital unions represent an important family form, cultural and institutional settings may promote childbearing within marriage. This means that measuring MS as marital unions partially conditions MS on RS (childbearing within marital unions) and makes the interpretation of Bateman's third principle problematic. Naturally, mating may also occur between persons who are not living together, and these interactions are difficult to measure on a large scale [27]. However, in the present study on contemporary Finland less than 5% of actual reproduction occurs outside of co-residing or marital unions [28]. We thus largely circumvent the issue pertaining to unobserved non-marital cohabitation by employing data that capture all cohabiting unions, whether marital or not.

### (b) Mating success as the number of unions and union duration

Several studies on humans have presented evidence against the notion that women do not benefit from multiple mates and that men should opt for a strategy that favours more partners to achieve higher fitness [29–31]. Among the Pimbwe of Tanzania, women who have multiple marriages have higher RS (at least if they marry more than twice), whereas the same is not found for men [32]. In her review of a range of human societies, Scelza [31] notes the many ways in which females can and do seek out multiple mates. Because humans are a species characterized by high parental investment, long childhood and facultative paternal care, the number of mates and the duration that one has access to a mate (or several) may shed light on different aspects of mating strategies. Leaving a current mate for the prospect of finding a new one is undertaken at the risk of lower cumulated mate exposure. Consequently, the fitness benefits each partner receives from staying an additional year with their current partner have to be greater than any benefits accrued from acquiring a new partner. The trade-off between mate quantity (additional births from other mates) and duration in union (continued investment in common offspring) may be essential. These claims have led to an interest in measures that capture exposure time to a mate [33,34].

In their paper, Borgerhoff Mulder & Ross [33] explore different components of MS on which sexual selection may operate among the Pimbwe of Tanzania. Exploring both number of marriages and marriage duration, they find that, the number of years with a spouse, not the number of spouses, predict RS more strongly for males than for females. This supports the notion that sex differences in fitness returns can operate via mating strategies other than the number of mates, argued for in Bateman's third principle [33,35]. In this study, we examine both mate number and the cumulated duration with any mate in a different socio-cultural context, namely present-day Finland.

### (c) Bateman's principles under social stratification

Whether to leave or to stay with a current mate may vary by mate quality and the mate's ability to invest in the common offspring. Individuals who possess traits that are desirable to others will also be more likely to find a new mate should they choose to leave their current one. In humans, one trait that is desired by men and women alike is resource access. Notwithstanding cross-cultural variation, mate choice data suggest a female preference for men of equal or higher status, while men do not show such status preferences to the same extent [36,37]. Consequently, in many societies men with low socio-economic status are more likely to have fewer children, to be childless [38], and to be disadvantaged in the search and competition for partners [39]. However, in our population of contemporary Finns, such disadvantages are found among both women and men with low socio-economic status, as they have shorter union durations and higher rates of childlessness than others [39,40].

Currently, little is known about whether the correlation between MS and RS is heterogeneous with respect to social status. Jokela *et al*. [23] find a stronger (marital) Bateman gradient among Black men than White men in the US, and suggest that this reflects underlying group differences in socio-economic status and adult sex ratios. Fieder & Huber [41] analyse the association between the probability of ever being married and RS in the contemporary USA, and find that higher wages positively predict RS because a higher proportion of high-earning men marry [41]. This finding corroborates patterns found in earlier studies from industrialized nations predicting childlessness [42]. Whereas studies on the effect of MS on RS across social strata are rare, a number of analyses on social status and RS show that men's (or husbands’) status positively predicts RS or negatively predicts childlessness, whereas this is not the case for women [43–49]. Here we test whether Bateman's principles hold across an entire national population, or whether they vary with different income for men and women. See tables S1–S3 for further description of the sample.

### (d) Study population

Finland provides a useful context to study Bateman's principles in an industrialized high-income society. It has a high degree of serial monogamy, divorce, non-marital cohabitation, and non-marital childbearing [50]. No-fault divorce is practised, and relatively high separation rates make childbearing with multiple mates common. Female labour market participation is high and both mothers and fathers partake extensively in childrearing, although women typically devote more time to childrearing than men, as indicated by mothers taking most of the parental leave [51]. These demographic behaviours and gender relations are typically associated with Nordic countries, but have been spreading across most industrialized countries for decades [52]. An examination of the Bateman principles in contemporary Finland—a socio-cultural context with a high degree of paternal investment and where both sexes are free to switch mates—will help to broaden our understanding of sexual selection in humans.

### (e) Aims and contribution

To the best of our knowledge, this is the first comprehensive test of Bateman's principles in a contemporary low-fertility population. Our study makes at least four important contributions. First, we capture both non-marital and marital co-residential unions, producing a more accurate measure of MS. While marriage is a precondition for reproduction in some societies in which the Bateman gradient has been explored, this is not the case in many contemporary industrialized, low-fertility societies. Second, by deploying national registers of the entire population, we avoid issues with statistical power, sample attrition, and under-reporting of male fertility or children from previous unions. Third, we compare MS operationalized as the cumulated number of unions and as cumulated union duration, respectively. In doing so, we build on previous work that has unpacked MS with duration data from a small-scale non-industrialized society [33]. Fourth, we are able to test whether any association between MS and RS varies across social strata. This is important, as social status is an important trait in mate selection and is associated with differential outcomes on the mating market and in childbearing [39].

## 2. Methods

### (a) Data

We use Finnish register data on Finnish-born individuals born between 1969 and 1972, alive in 2018 and who had resided in Finland since the year of their 18th birthday; in total 219 086 persons. We focus on the population who remained residents in Finland in order to prevent under-estimation of births and unions that might have occurred abroad. Data on non-marital cohabitation in the total population exist since 1987, the longest-running population-wide record in the world. Marriage data are also available for this period. Therefore, we can analyse non-marital and marital cohabitation and childbearing histories during the ages of 18–46 years of the 1969–1972 birth cohorts.

### (b) Mating success

We measure the cumulated number of unions by the age of 46 years from each unique union, that is, with the same partner. Non-marital cohabiting unions are defined by Statistics Finland. The definition considers a person to live in a cohabiting union if he or she is domiciled for more than 90 days with an opposite-sex individual who is not a sibling or parent, with an age difference below 20 years. The approach has been assessed in previous research in Finland [53,54] and elsewhere [55] and found to accurately capture cohabiting unions. It is nevertheless not possible to universally exclude non-sexual cohabitation of opposite-sex individuals who share a dwelling. To measure the cumulated union duration, we add the total number of years a person has spent in any union. A union that is first non-marital and thereafter turns into a marriage is counted as a single marital union. Union duration is measured from the first to the last dates of observation. To underscore the necessity of measuring non-marital unions, we demonstrate, in electronic supplementary material, table S4, that marriage-only measurement under-estimates the share of those ever in one union, and greatly under-estimates the share of those ever in two or more unions.

### (c) Reproductive success

RS is measured as the cumulated number of children ever born to a parent by the age of 46 years. Male and female fertility is identified using this parent–child linkage. Paternity is acknowledged for a spouse and by consent among the non-married. Contested or non-confirmed paternity is investigated vigorously by social services, and registers are updated accordingly. Only about 2% of births lack a father-link in the records. Electronic supplementary material, figure S1 shows how much male fertility is under-estimated owing to our cut-off point being age 46. For the 1963 cohort, which we can follow up to age 55, fewer than 1.5% of all births for males by age 55 occur after age 46.

### (d) Social status

Our main measure of an individual's social status is income rank, which is based on earnings, capital income, and social transfers that are conditioned on earnings. We use the maximum value of income in any of the calendar years when a person was aged 44, 45 and 46 years, and create rank centiles. This approach prevents underestimation driven by temporary declines or breaks in employment.

### (e) Analytical strategy

To analyse Bateman's first principle—that men have higher variance than women in RS—we calculate ‘the opportunity for selection’, *I*, separately for men and women across five-centile income rank averages, where *y* is the number of children born to a parent by age 46, $\sigma_{y}^{2}$ is its variance, and ${\bar{y}}^{2}\;$is its squared mean [29,56].

2.1 $$I=\frac{\sigma_{y}^{2}}{{\bar{y}}^{2}}.$$

To analyse Bateman's second principle—that men have higher variance than women in MS—we calculate ‘the opportunity for sexual selection’, *I*_S_, separately for men and women across five-centile income rank averages, where *u* is the cumulated number of unions by age 46, $\sigma_{u}^{2}$ is its variance, and ${\bar{u}}^{2}\;$ is its squared mean.

2.2 $$I_{S}=\frac{\sigma_{u}^{2}}{{\bar{u}}^{2}}.$$

For completion, we use the same equation to describe variance in the cumulated union duration by age 46.

The stochastic variation in MS increases with the OSR and decreases with the population size [18]. To the extent that sex ratios and population size vary across social strata, differences in *I* and *I*_s_ across sex and social strata may result in a spurious relationship between social strata and mating success. However, our population subgroups are by default quartiles of the full population and are assumed to share the same mating pool, and the sex ratio is about 1.05. Importantly, the underlying model for differences in mating success across income quartiles implies (a) competition over mates, and/or (b) differential life-history strategies [11]. Regardless, *I* likely contains an unknown spurious component. The Finnish population does not display mate domination in that it practises (serial) monogamy, and has comparably low mean fertility of about 1.8. These two factors decrease rather than increase the general influence of sexual selection. Electronic supplementary material, table S6 report all components of *I*—mean fitness, variance and squared mean fitness—across income groups, and shows also *I* when mean fitness is held constant at the sex-specific population average.

Lastly, we analyse Bateman's third principle—that MS is more positively associated with RS for men than women. We fit linear regression models and estimate the association between standardized MS and standardized RS, for men and women separately, across income quartiles, adjusting for birth cohort. We repeat this exercise for the full population and the population who had at least one union by age 46. This distinction helps us to separate the effect of ever having a union on RS, on the one hand, and the effect of the number of unions on RS, on the other hand. Regressions are estimated separately for each operationalization of MS (continuous measures of union count and union duration) as the dependent variable:

2.3 $$\mathrm{RS}=\beta_{0}+\beta_{1}\mathrm{MS}+\beta_{2}\mathrm{Cohort}.$$

In electronic supplementary material, figures S4–S8, we further describe the patterns of mating and reproduction in our population by showing the relationship between MS and RS at each union count for men and women. MS is there estimated on an ordinal scale, holding one union as the baseline level for the cumulated number of unions. This exercise complements the results on the relationship between MS and RS that have focused on point estimates rather than MS as the discrete number of unions. To accommodate the count distribution of *y*, we estimate Poisson regressions, with the predicted mean of the associated Poisson distribution given by

2.4 $$\begin{array}{rl} E(y|x) & =\exp(\beta_{0}+\beta_{1}\mathrm{MS}+\beta_{2}\mathrm{Sex}+\beta_{3}\mathrm{Income}\\ & \;+\beta_{4}(\mathrm{MS}\times \mathrm{Sex}\times \mathrm{Income})+\beta_{5}\mathrm{Cohort}), \end{array}$$

where *x* refers to regressor variables and *β* parameters to be estimated. When we analyse MS as union duration, we categorize union duration into 5-year bin categories, where 10–14 years in a union by age 46 is the baseline level. Here, we include an interaction of MS, sex and income quartile, which is the regressor variable of specific interest. We report the average marginal effect (AME) of each cumulated union count by age 46:

2.5 $$\mathrm{AME}=\frac{1}{N}\sum \frac{\partial E[y_{\mathrm{RS}}|x_{\mathrm{MS}},w]}{\partial x}\beta_{k}.$$

This can be interpreted as the difference in the mean number of children $y_{\mathrm{RS}}$ compared with the baseline level, associated with each specific cumulated union count $x_{\mathrm{MS}}$ after conditioning on the values of covariates *w* over the population *N*. We also report the AME of each cumulated union count adjusted for union duration, and the AME of union duration adjusted for accumulated union count.

## 3. Results

### (a) Bateman's first and second principles

**Figure 1.**
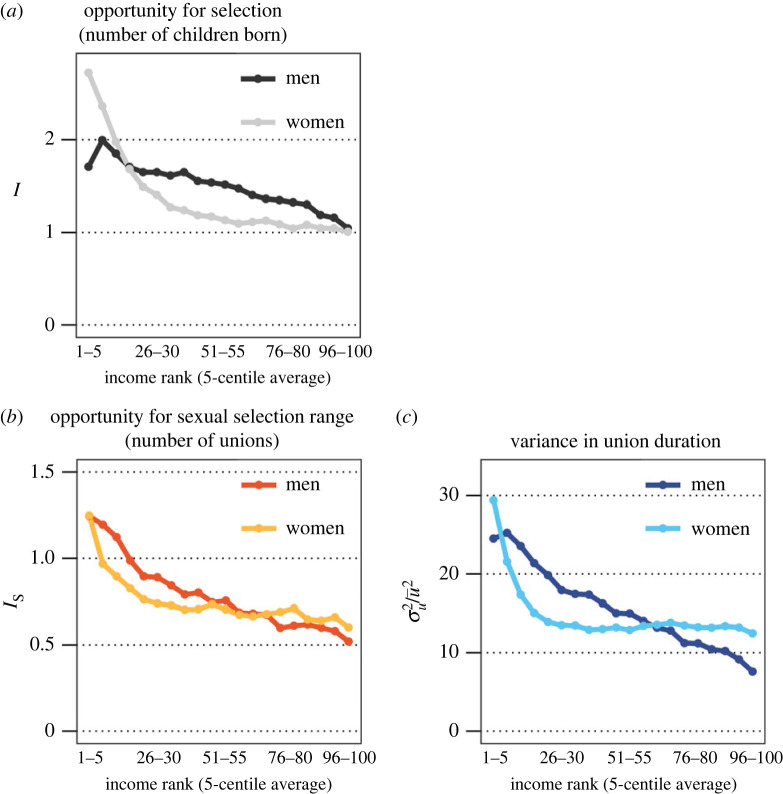
(*a*) Variance in number of children across income rank for men and women (opportunity for selection, *I*). (*b*) Variance in cumulated number of unions across income rank for men and women (opportunity for sexual selection, *I_S_*). (*c*) Variance in cumulated union duration across income rank for men and women. Income rank is categorized into five- centile averages.

Figure 1*a* shows the opportunity for selection (variation in RS, *I*) across income rank. For both sexes, *I* is largest at the lower end of the income rank distribution (men 1.71, women 0.68, at the 25th centile). On average, men have higher variance in *I*, except at the lowest and highest income ranks. Figure 1*b* show the opportunity for sexual selection (variation in MS, *I*_S_) as defined by the cumulated number of unions. *I*_S_ is lower at higher income ranks. Males in the lowest income quartile have a higher *I*_S_ than females (0.9 versus 0.7, respectively, at the lowest income quartile), while males in the highest income quartile have somewhat lower *I*_S_ than women (0.60 and 0.68, respectively, at the third income quartile). Figure 1*c* measures the coefficient of variance for union duration. In terms of sex differences the pattern is quite similar to that of figure 1*b*.

### (b) Bateman's third principle

**Figure 2.**
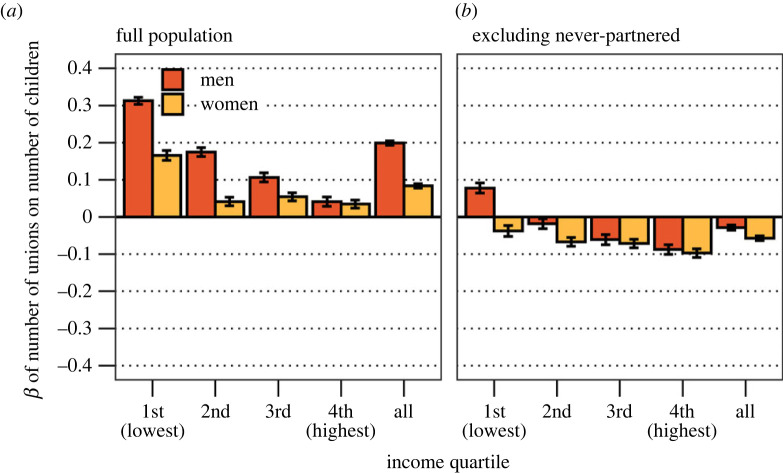
(*a*) Estimated effect of cumulated number of unions on number of children across income quartile for men and women, full population. (*b*) Estimated effect of cumulated number of unions on number of children across income quartile for men and women, never-partnered excluded. Both models adjusted for birth cohort. Standardized coefficients.

**Figure 3.**
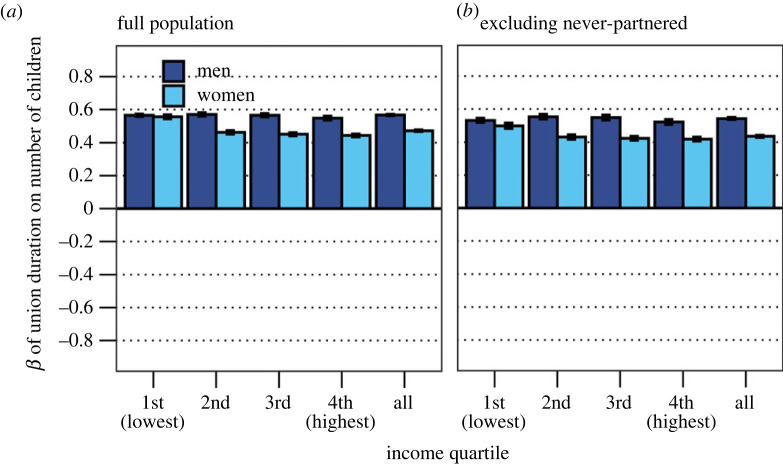
(*a*) Estimated effect of cumulated union duration on number of children across income quartile for men and women, full population. (*b*) Estimated effect of cumulated union duration on number of children across income quartile for men and women, never-partnered individuals excluded. Both models adjusted for birth cohort. Standardized coefficients.

Figure 2 shows the main effect of MS (cumulated number of unions) on RS (the number of children). We estimate this effect separately for each income quartile of the population, and results for each income quartile subgroup are presented along the *x*-axis. Figure 2*a* shows that the number of unions positively predicts RS for both women and men. This effect is stronger for men than women (on average, *β* is 0.157 for men and 0.061 for women). The sex difference is lower at higher income ranks, and in the highest income quartile there is no sex difference. Figure 2*b* reiterates this exercise by excluding individuals who have not had any union by age 46, who amount to about 8% of the population. When never-partnered are excluded, a higher number of unions predict fewer children for both men and women. The exception is men in the lowest income quartile, for whom *β* is 0.63. The difference as compared with the models that include never-partnered suggests that the interrelation between MS and RS is largely driven by whether persons have had at least one union, and indicates that the relationship may be nonlinear. Figure 3 measures the association between cumulated union duration and RS. Union duration positively predicts the number of children, and more strongly so for men. The positive gradient as well as the sex-specific patterns remain when considering only those who had at least one union (figure 3*b*).

### (c) Additional specifications and robustness checks

Because we are interested in the full variance in RS, our main models include individuals who have never had a union as well as individuals with no children. Age at first birth, given that a birth has taken place, is considered an endogenous aspect of mating strategies. In electronic supplementary material, figures S2 and S3, we estimate the linear relationship as suggested by Bateman's third principle, excluding childless individuals and adjusting for age at first birth. We then find a negative association between number of unions and RS on average, while a modest positive effect remains for men in the lowest income quartile. Union duration positively predicts RS after adjusting for age.

We have examined how childbearing at later ages influences the results by simulating increased male childbearing corresponding to a 1.5% increase in births from age 46, which is an upper bound as indicated by previous research on male fertility [57,58]. We have here assumed that these births occur among men who have had multiple unions. Electronic supplementary material, figure S4 shows that these additional births do not change the observed patterns, and in particular they do not produce a positive Bateman gradient, except in the case of low-earning men, as we have already observed.

Moreover, we have made the most out of our population-wide data in order to describe the nonlinear effect of MS on RS (electronic supplementary material, figure S5). The negative association between the distinct number of cumulated unions and RS is stronger in higher-income quartiles than in lower ones. Men in the lowest income quartile constitute the only group for which the number of unions has a positive association with RS. For example, for these men, having had five or more unions by age 46 compared with one union is associated with almost 0.5 more children, while for women, the corresponding estimate is practically zero. Conversely, in the highest income quartile, having had five or more unions compared with one union is associated with about 0.5 fewer children for both sexes. The marked differences across income quartiles are less strong when RS is predicted by cumulated union duration (electronic supplementary material, figure S5B1–B4). For longer union durations, the association with RS is stronger for men than for women in all income quartiles, while for shorter durations only in the third and fourth income quartiles. Adjusting for union duration removes the impact of the number of unions (electronic supplementary material, figure S6). Negative binomial models (electronic supplementary material, figure S7) have been used to check that the comparable Poisson models (electronic supplementary material, figures S3 and S4) do not suffer from overdispersion. Estimates from the Poisson and negative binomial models are virtually identical. The positive association between cumulated union duration and number of children remains after controlling for the number of unions. Union duration and number of unions are highly correlated (*r* = 0.419 for men and *r* = 0.262 for women, in electronic supplementary material, table S5), which offers support for the importance of mate retainment and mate exposure. Finally, as discussed, most previous work in the literature relies on marital partners only when operationalizing MS [23]. For comparability with these studies, we have analysed Bateman's third principle using only marital unions and duration in marriages (electronic supplementary material, figure S8). These results are congruent with the Bateman gradient, but not conclusively.

## 4. Discussion

This is the first study to our knowledge to assess Bateman's principles in an industrialized high-income society using population-wide data, and to include non-marital cohabiting unions. Building on recent efforts that expand the concept of MS [33,34], we analyse union duration in addition to the number of unions. Importantly, we consider that mating strategies vary by mate quality as measured by social status [59]. By contrast to most previous research, we consider not only marital but also non-marital cohabitations. Our data support Bateman's first principle: there is, on average, higher opportunity for selection (*I*) for men than women in contemporary Finland. The results also indicate that Bateman's second principle holds: men have on average higher opportunity for sexual selection (*I*_S_) as measured by the cumulated number of unions. Men also have on average higher variance in years of cumulated union duration than women. However, we demonstrate that these relationships shift depending on social strata: the opportunity for selection and sexual selection are higher among men than women at lower levels of income, but at higher levels of income, men have lower opportunity for selection than women.

Bateman's third principle is not universally supported. In the population as a whole, data suggest a positive relationship between number of unions and RS, and that this association is higher for men than women. However, we show that this relationship is driven by ever forming a union rather than the number of unions—as recently found by measuring marriages in the US [41]. Among the ever-partnered, who constitute 92% of the total population by age 46, there is no positive effect of additional unions. This pattern is noteworthy; while it stresses sex differences in mating, the Bateman gradient concerns (continuous) mate acquisition across the life course, not a dichotomous relationship of ‘ever mating’ or not.

We further show that, among men and women who have had at least one union, each additional union is negatively associated with RS (compared with having a single union). Notably, this relationship differs across social strata. For men (but not women) in the lowest income quartile, having multiple unions positively predicts RS. Moreover, we explore how union duration correlates with RS. Here, we find a positive association between union duration and RS, which is higher for men than women consistently across social strata. We also show that the positive relationship between having a single union and RS disappears for both men and women when adjusting for cumulated union duration. This indicates that having (one) union leads to RS through mate exposure.

Overall, these data lend little support for the argument that seeking more mates universally increases the number of children more for men than women. However, in line with Borgerhoff Mulder & Ross's examination [33] of the Pimbwe, we find that men incur somewhat higher fitness benefits than women from longer exposure to a mate in pair-bonding. This observation aligns with hypotheses that emphasize the value of mate quality and mate retainment [33,60]. Thus, application of the Bateman principles to humans may provide an incomplete story of mating strategies, partly because it discounts union duration. Because Bateman's original idea pertains to number of mates, and not mate duration, confusion may arise if MS were to be operationalized as number of years with a mate. Nevertheless, scholars may consider that fitness returns may, under some circumstances, increase via long-term access to one or several mates, rather than only through a high number of mates. Additionally, our analysis suggests that men in the lowest social strata are the only group who consistently benefit from serial monogamy. This indicates heterogeneity across social strata in the pay-offs to mating strategies.

To interpret the observed patterns, it is important to understand the socio-ecological context in which these mating strategies play out. In contemporary Nordic societies, cultural norms dictate that fathers are active in childrearing. High female labour market participation and social and family policies make childbearing less financially costly to the individual, and women less dependent on a male provider. This may lead both men and women to be choosy in their choice of partner, and once in a union focus on mate retainment, rather than seek additional mates. So-called ‘typical’ male or female mating strategies may be less beneficial in this and similar populations. Nevertheless, data suggest that Finnish men with the lowest income are the least likely to have long-term partnerships and are disadvantaged in the mating market. Finland is a society in which union dissolutions are ubiquitous, and low-status men in particular face poor prospects of retaining a mate. Our data indicate that, among the low-income population, having multiple mates is associated with higher RS.

We acknowledge that in this contemporary Finnish population, which has a high life expectancy and low birth rates, fitness consequences of mating strategies may be different from those in natural fertility populations. Yet, measuring fertility in industrialized human populations is important and adds to our understanding of behavioural strategies [61]. Recent genetic and phenotypic findings suggest that natural selection may operate in contemporary human populations, and that variance in reproduction over phenotypes correlates with status seeking [62–64]. We have applied measures of *I* and *I_S_*, and the significance of these parameters for selection and sexual selection is contingent on several critical assumptions, such as homogeneity in the random component of RS and MS across sex and income subgroups [15,18].

It is difficult to ascertain whether the difference between our findings and previous research relates to methodology and data quality, and/or socio-cultural differences. The question of whether or not to measure MS through marriage only, or also to include non-marital partners, depends on whether childbearing is confined to marriage in the population under study. MS as spouses only, is likely a valid strategy for nineteenth-century Finland [21], but not for contemporary Finnish society, or for modern-day White and Black ethnic groups in the US, where the latter have exceptionally high rates of non-marital cohabitation [23]. We acknowledge that we do not capture mates who have never resided together/been registered at the same address. A more valid measurement would be achieved if non-cohabiting mates were also included. However, in societies like Finland, almost all childbearing takes place in co-residential unions [65], which makes our case a novel contribution to research on humans. By avoiding conditioning on marital status, we at least circumvent built-in reverse causality of MS on childbearing. Future work may factor in the age of mates, age at childbearing and other measures of mate quality to understand various components of MS [33].

## Data Availability

The code used to process raw data to produce all output in this study, the processed data output and the code to visualize this processed data as it appears in the manuscript are all provided in the electronic supplementary materials [66]. The raw data draw on individual-level population-wide registers, which we are prohibited by law to use outside secure servers provided by Statistics Finland. However, researchers may apply for and purchase register data themselves. Contact the corresponding author for further information.
